# Long-term surveys of ungulates’ effects on tree and shrub species in mountainous forests –outcomes and potential limits

**DOI:** 10.1007/s00267-024-02063-4

**Published:** 2024-10-24

**Authors:** U. Nopp-Mayr, W. Lechner, S. Reimoser, F. Reimoser

**Affiliations:** 1 Institute of Wildlife Biology and Game Management, Department of Integrative Biology and Biodiversity Research, University of Natural Resources and Life Sciences, Vienna, Gregor-Mendel-Str. 33, 1180 Vienna, Austria; 2 Research Institute of Wildlife Ecology, Department of Interdisciplinary Life Sciences, University of Veterinary Medicine, Savoyenstraße 1, 1160 Vienna, Austria

**Keywords:** Herbivory, Woody plants, Diversity indices, Hill numbers, Exclosure-control pairs, Height growth

## Abstract

Ungulate herbivory might induce different effects on the diversity and growth of trees and shrubs. The density, distribution, and the species of ungulates as well as plant communities’ composition and other factors determine whether ungulate herbivory promotes or limits plants’ diversity and growth. The impacts of ungulates on woody plants are commonly surveyed with exclosure-control approaches. In practice, such surveys frequently only cover short periods of time, addressing immediate management needs. Long-term surveys, documenting lasting effects of ungulate herbivory, are highly needed, but still rare. However, the general transferability of outcomes of long-term surveys might be limited due to different disturbing factors. This study addresses two basic aspects of long-term monitoring in mountainous forests, based on a unique 30-year data set: (1) Possible long-term effects of herbivores on forest vegetation (e.g., species/structural diversity of woody plants) and (2) potential differences between short-term and long-term surveys in terms of height growth patterns. In our study, diversity of woody plant species showed great variability with no significant impact of ungulate herbivory. The presence of ungulates had a significantly negative effect on the vertical structural diversity and growth of trees. Due to the slower growth on control plots, it took trees longer to reach a 160 cm height-threshold with their terminal shoots than on exclosure plots. Our long-term control-exclosure data set indicated that long-term survey data indeed might differ from growth patterns represented by short-term surveys. This can be induced by several factors, like site-specific growth patterns of trees, occurrences of natural abiotic disturbances that influence the functional life of exclosures, and others.

## Introduction

Herbivory, defined as the consumption of living plant tissues by animals, comprises a number of different processes like seed predation, browsing, grazing, mining, or bark peeling (Schowalter 2016). It is one of the most crucial disturbances within an individual plant’s ontogeny (Aguirrebengoa et al. 2018; Massad 2013; Selaković et al. 2017) and has major impacts on species composition and biodiversity of plant communities and ecosystems (Bernes et al. 2018). Starting with seedling herbivory, herbivores shape landscapes as their preference for certain seedlings leads to cascading changes in plant species communities, biodiversity, and forest regeneration (Cilles et al. 2016; Williams and Brodie 2023). The effects of leaf browsing and grazing on plants range from differences in morphology (Persson et al. 2005) to changes in metabolism (Gaquerel et al. 2014; Schrijvers-Gonlag et al. 2020; Zhou et al. 2015) and alterations in interspecies competition (Sebata 2013). These small-scale effects can in turn lead to changes in species compositions of plants and animals (Wisdom et al. 2006), nutrient availability, and nutrient cycles (Abbas et al. 2012; Murray et al. 2013).

In temperate forest ecosystems, ungulates are one of the most impactful herbivores (VanderMolen and Webster 2021; Weisberg and Bugmann 2003). Ungulates prefer certain plant species or plant individuals to others due to their availability, accessibility, nutrient value, defense status and other factors (Holeski et al. 2016; Ingram et al. 2013). The selective feeding by ungulates can tip the scales in the inter-species competition of plants to favor one side or the other (Maxwell et al. 2019), as herbivory can lead to a complete disappearance of seedlings and smaller plants or reduced height growth of saplings and bigger individuals (Beguin et al. 2016; Hood and Bayley 2009). These effects can be exacerbated through inherently slow annual height growth due to environmental factors such as soil fertility (Zamora et al. 2001). Selective feeding can lead to changes in species composition, productivity, and biodiversity of plant communities (Royo and Carson 2022), which in turn might result in a change in animal communities (Seager et al. 2013). The extent of related changes also depends on environmental drivers (e.g., aspect or light availability), potentially reducing or amplifying impacts of ungulates on the vegetation (Kupferschmid et al. 2014, 2020).

The impacts of ungulate herbivory on trees have been the subject of many surveys, with exclosure-control settings being the most common approach (Bellingham and Allan 2003; Casabon and Pothier 2007; Millett and Edmondson 2013; Pellerin et al. 2010; Reimoser et al. 1999, 2023; Russell and Fowler 2004; Tschöpe et al. 2011). By comparing vegetation data from a fenced plot to which the ungulates have no access, with vegetation data from an accessible control plot ungulates’ impacts can be separated from other driving factors. This allows for conclusions on the effect of ungulates on biodiversity, height growth, forest regeneration, species composition, and more (Hedwall et al. 2018; Kay and Bartos 2000; McGarvey et al. 2013; Perkovich and Ward 2022; Seymour et al. 2016).

As the most vulnerable part of a tree’s life cycle for ungulate herbivory lies in its early years, many exclosure studies particularly address these early stages and related community responses. However, the complexity of plant-animal interactions together with intra- and interspecific interactions between plants/species makes it difficult to translate recent influences of wild ruminants into impacts at later developmental stages. Compensatory mortalities, gap-forming and gap-filling dynamics, human intervention as well as interactions with abiotic drivers might play a decisive role. As a consequence, lasting effects of ungulate herbivory cannot be adequately estimated (Nopp-Mayr et al. 2020), when only covering the first years after germination. In practice, herbivore impact surveys frequently cover only short periods of time, addressing immediate management needs. Long-term surveys, documenting lasting effects of ungulate herbivory are highly needed, but still rare. However, outcomes of long-term surveys might only be representative for specific environmental settings and logistic situations, which allow for such a monitoring over decades. The latter particularly applies for long-term monitoring done by forest practitioners that primarily aims at providing data for ongoing ungulate management decisions yearly or in short periods of few years.

Our study was based on a regular state-wide ungulate herbivory monitoring system in the westernmost state of Austria (Vorarlberg), which provides a unique data set of 30-year surveys. It is the longest lasting statewide ungulate impact survey in Central European mountainous forests with a large number of sample plots. Within this system ongoing ungulate impacts and their lasting effects on forest regeneration are monitored and evaluated, based on comparisons of ungulate exclosure plot data with data from open control plots accessible for ungulates. There are plot pairs, which have been recorded for the last 30 years having their fence intact the whole time.

The survey allowed us to explore potential advantages of long-term data sets, capturing the complex cascades of competition, compensatory mortality and more at later developmental stages, which would have been missed in the course of short- or mid-term surveys. This included the following questions that are relevant for forest and ungulate management issues and frequently not addressed to this extent or in a comparable combination. Nevertheless, the complex effects of herbivores on different woody taxa or vegetation strata in terms of species and structural diversity require their simultaneous consideration: (1) Are effects of ungulate herbivory in terms of tree or shrub species diversity detectable after a 30-year time span? (2) Is the structural diversity (in terms of height class diversity of trees) different in the fenced plots compared to the control plots after 30 years? (3) Do plot types (open access vs. exclosure) differ in height class distribution of trees on the long term? (4) Does the number of years until terminal shoots of trees outgrow the height of ungulate browsing differ between the fenced plots and the control plots? (5) Do long-term data particularly represent sites with lower height-growth performance, when they are provided within a monitoring scheme supporting ungulate management needs?

## Materials and methods

### Study area

**Fig. 1:**
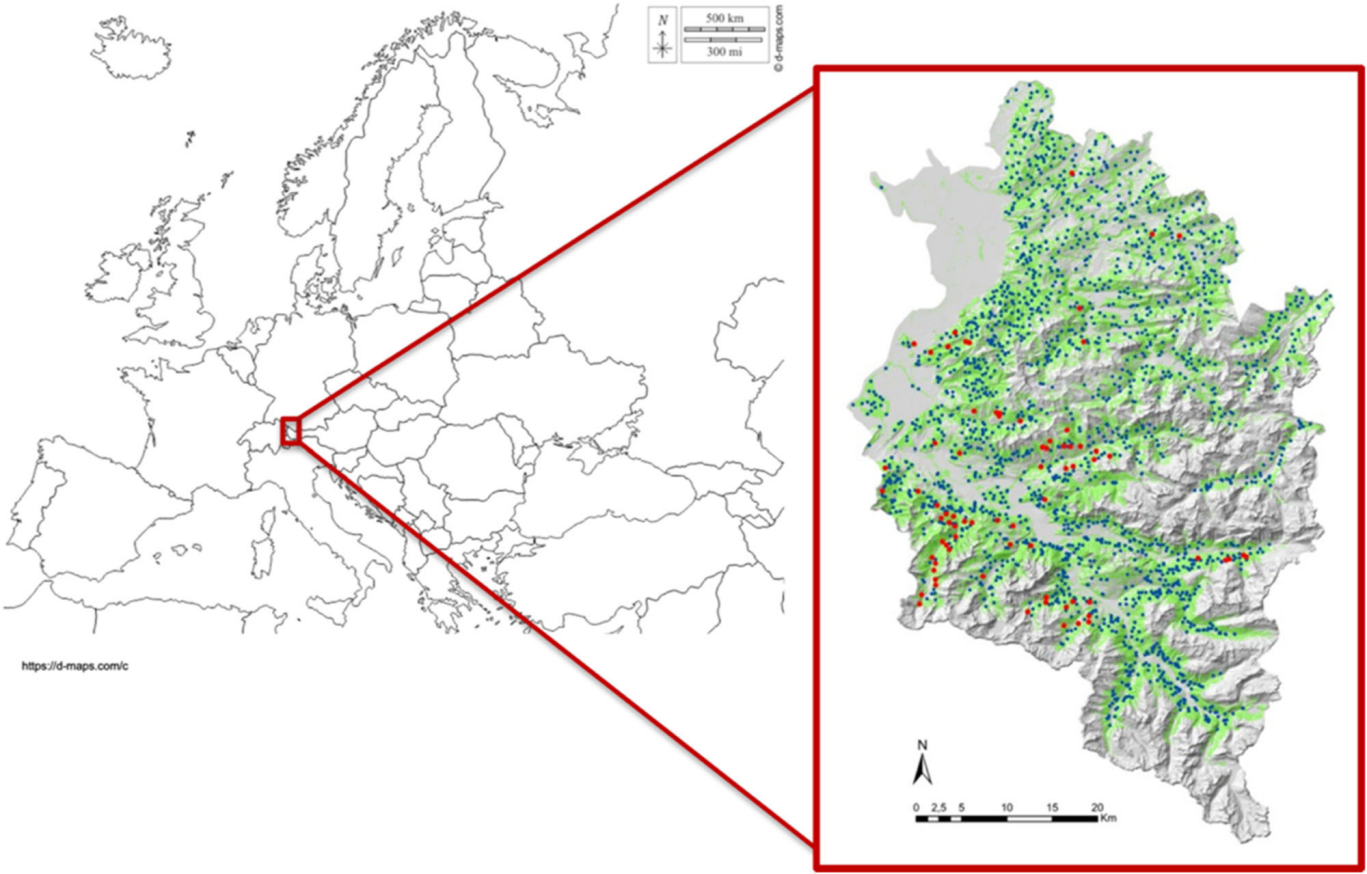
Location of the study area and distribution of investigated survey plot pairs since 1989 (*n* = 2528, blue dots) and during the last survey 2022 (*n* = 73, red dots)

Forest regeneration was monitored in the westernmost state of Austria (Vorarlberg) with a proportion of forest cover of 36% (Fig. 1). The state’s geology changes distinctly along a north-south gradient from the Molasse and Helvetic sediment zones to a Flysch zone, then the Northern Limestone Alps, and in the south the crystalline rocks of the Central Eastern Alps. The annual mean temperature in the study area ranges between 6 and 9 °C and the mean annual precipitation in the surveyed areas lies approximately between 1.200 and 2.400 mm. The monthly precipitation pattern shows a maximum in June although in some areas a second maximum in November is typical. The duration of snow cover varies strongly between survey plot pairs with 50 days for plots closer to the valley bottoms to 180 days in areas around 1.500 m above sea level. The elevation range, covered by plot pairs, reaches from 500 m a.s.l. to approximately 1.700 m a.s.l.

The plot pairs are assigned to different representative forest communities: (i) Submontane deciduous forests with dominating European beech (*Fagus sylvatica*), frequent European ash (*Fraxinus excelsior*) and sycamore maple (*Acer pseuodoplatanus*); Norway spruce (*Picea abies*) and silver fir (*Abies alba*) often promoted through silviculture. (ii) Montane mixed forest communities consisting mainly of Norway spruce, silver fir, and European beech, with European ash, sycamore maple, and wych elm (*Ulmus glabra*) mixed in. (iii) Upper montane forests (mainly Norway spruce and silver fir). (iv) Subalpine Norway spruce forests at higher altitudes with Mountain ash (*Sorbus aucuparia*) mixed in. In Vorarlberg, most forests are managed with silvicultural systems aiming at permanent forests without clearcuts, as approximately 50% of the state’s forests are protection forests against avalanches, rock falls, and erosion. The most common type of permanent forest is the selection forest.

Ungulate densities are high, with the most common ungulate species being red deer (*Cervus elaphus*), roe deer (*Capreolus capreolus*), and chamois (*Rupicapra rupicapra*). The state tries to keep the flat terrain in its northern and north-western parts free of red deer. Yearly hunting bags are on average 1 red deer, 2 roe deer, and 1 chamois per km^2^ (based on a hunting season from May to December, see also Table 1).

**Table 1:** Average yearly hunting bags (individuals/km^2^) of the main three ungulate species within two different time periods. Minimum and maximum hunting bags within the entire time horizon (1985–2022) are given for the respective years

Period or year	Hunting bag (n/km^2^)		
	Red deer	Roe deer	Chamois
1985–1994	0.7	1.7	0.6
2015–2022	1.1	2.0	0.5
Min. year	0.5 (1990)	1.5 (1989/90)	0.3 (2007)
Max. year	1.3 (2013)	2.3 (2010/12)	0.7 (1987)

### Field sampling

With an amendment in Vorarlberg’s hunting regulations in 1988, the calculation basis for the annual game harvest plan changed from imprecise ungulate counts to the extent of damages caused by ungulates. To monitor ungulate damage, pairs of fenced plots and unfenced control plots were established in the state’s forests and were to be surveyed every 3 years. In total 3405 pairs of ungulate exclosures and unfenced control plots were established at altitudes between 500 and 1.100 m a.s.l. since 1989, the last survey was in 2022. Until this point of time, many fence or control plots had been destroyed or disturbed by natural abiotic disturbance events like storm throw, snow loading, or rock falls. Plots that experienced other disturbances than ungulate herbivory were omitted. Thus, a final number of 73 plot pairs could be surveyed over the entire 30-year time span.

The first survey ( = time of plot establishment, year 0) and the second survey (year 3) were carried out by F. Reimoser and team, the last survey 2022 by W. Lechner and F. Reimoser. The years in between were sampled by forest supervisors and many plot pairs had been (unplanned) abandoned by them after several surveys, when a certain tree height was reached.

The guidelines of the state Vorarlberg for the monitoring of ungulate impacts/damages via plot pairs define bindingly how and where plot pairs are to be created (see also Reimoser et al. 2023). They must be installed on areas where forest regeneration is in an early stage (saplings beneath 50 cm in height) or is expected to start soon. Canopy cover needs to be beneath a threshold (ca. 80%) that allows for development of seedlings on the ground after germination depending on forest community and altitude.

The plot pairs contained only natural forest regeneration and no planted trees. The forests in which the plot pairs were located are all subject to forest management. The vegetation within the plots however was not tampered with. Plots that had been directly affected by forest management were not sampled. If the regeneration of a plot pair was influenced unequally between a fenced and paired unfenced plot by forest operations in the mature stand, this plot pair was omitted from the investigation. The entire sampling procedure was optimized to capture effects of ungulate herbivory and to keep other driving factors as constant as possible.

The fenced plots were designed to keep out larger (ungulate) herbivores (i.e., red deer, roe deer and chamois). Smaller herbivores like the European hare (*Lepus europaeus*) or rodents still had access to the fenced plots. To ensure comparability within a plot pair, plot sites were chosen with highest possible similarity in environmental conditions. Which of the two plots was fenced was determined at random by a coin toss. Exclosure plots were surrounded by a 2 m tall mesh fence, which enclosed an area of 6 × 6 m. Plots were numbered and marked with wooden stakes in the middle and the corners. The fenced plot and the control plot were located at distances between 5 and 40 m. Larger distances would have reduced comparability within a pair, whereas limiting the distance to a shorter range than approximately 40 m would have increased the difficulty of finding two suitable and similar spots, which is especially challenging in steep and uneven alpine terrain. To eliminate edge effects, we surveyed a 5 × 5 m area within the 6 × 6 m zone of each exclosure and control plot.

Following the gap-maker–gap-filler approach (Lertzman 1992), we dynamically recorded the height of the largest tree individuals per tree species on the paired plots (open access and ungulate exclosure, each 25 m^2^), as this allowed us to assess herbivore impacts on those individuals, which have a high probability of attaining later developmental stages (Nopp-Mayr et al. 2020). We surveyed the six highest individuals per tree species and plot or, in case of lower stem numbers ( < 6), all occurring individuals per tree species and sampling plot (see Nopp-Mayr et al. 2020). We used the following height classes: 10 cm, 11–25 cm, 26–40 cm, 41–70 cm, 71–100 cm, 101–130 cm, 131–160 cm, 161–200 cm, 201–250 cm, 251–300 cm, … (continuing 50 cm classes). It has to be noted that the survey scheme per se does not provide for a termination of the surveys when a certain tree height (e.g. 2 m) is reached. The height classes cover an increasing height range with increasing height, starting with 15 cm intervals for seedlings and ending up with 50 cm intervals at later stages. These unequal class ranges were chosen for two reasons: (1) They account for typical height growth patterns in tree species with steep slopes at early life stages and flattening curves after decades (Manso et al. 2021); (2) consequently, small differences in height or height growth might be more meaningful in terms of competitive power and survival in smaller trees compared to later stages (Adams et al. 2007; Matisons et al. 2020; Walters et al. 2020). For shrubs, we visually assessed the coverage (%) of shrub species per survey plot, as individual entities and related numbers might not be easily identified in the course of a field survey.

No animal experiments were carried out. The ungulates were neither caught nor influenced by medication. They were only denied access to small patches within forests by means of a fence. The fencing was carried out in accordance with all relevant guidelines and regulations.

### Data preparation

We used the occurrence of different height classes to analyze long-term differences in the structural diversity of forest regeneration induced by ungulate herbivory (Nopp-Mayr et al. 2023). As this does not represent actual differences in absolute height growth between the exclosure and the control plots we further addressed height differences of the highest 6 individuals per tree species. To explore differences in diversity of shrub species, coverage data per shrub species was used as representative of species occurrence.

**Fig. 2:**
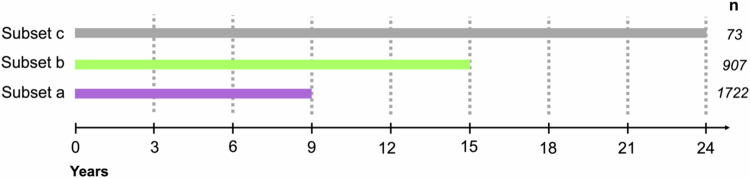
Three data subsets (a, b, c) with their investigation periods (9, 15, 24 years) and corresponding numbers of plot pairs (*n*) for which the share of plots, in which the tree species reached the height threshold of 160 cm, was analyzed every three years (year 3, year 6 etc.). Each subset comprised only the number of plot pairs that were recorded over the entire investigation period of the related subset (i.e., 9 years for subset a, 15 years for subset b and 24 years for subset c)

To address the question whether ungulates impact the time a tree species needs to grow out of the ungulate’s feeding range, we focused on the highest tree per tree species recorded in a 3-year cycle. The height, at which ungulates are assumed to have little to no impact on tree height growth was set to 160 cm. This height was chosen as red deer, the tallest of the three occurring ungulate species, very rarely feed on vegetation above this height (Renaud et al. 2003). To highlight effects of short-term vs. long-term surveys in terms of height growth performance, we created different data subsets, each covering different investigation periods (subset a: 9 years; subset b: 15 years; subset c: 24 years; see also Fig. 2). Each subset comprised only the number of plot pairs that were recorded over the entire investigation period of the related subset. Thus, the number of plot pairs decreased with increasing investigation period (Fig. 2). Per subset we calculated the share of plots, where a tree species reached a height of 160 cm in percent of the total number of surveyed plots, where the tree species occurred. These percentages were contrasted between the open access control plots and the exclosures.

### Statistical analyses

According to Chao et al. (2020), woody plants of the same species tend to aggregate spatially, potentially violating the assumption of individuals being independent sampling units. Therefore, the abundance data of trees and shrubs were transformed into incidence-based ( = presence/absence) data, which, in a species-by-sampling-unit incidence matrix, can be used for incidence-based diversity analyses. Accordingly, height data of the six tallest tree individuals per species and plot was transformed into an incidence-based height class dataset. This means that each plot is handled as an independent sampling unit (Chao et al. 2014; Chao et al. 2020).

To compare the impacts of ungulates on the diversity of woody plants and height classes, diversity estimates are needed. The first three Hill numbers of order q were chosen as these are commonly used diversity estimates. Hill number *q* = 0 refers to species richness and is henceforth referred to as “species richness”. Hill number *q* = 1, the exponential of Shannon’s entropy index, is henceforth referred to as “Shannon diversity” and *q* = 2, the inverse Simpson´s concentration index, is henceforth referred to as “Simpson diversity” (Chao et al. 2014). A major difference between the three mentioned Hill numbers is how much weight is attributed to rare species. Species richness is calculated with equal weight for rare species and common species because their detection probabilities are not accounted for. Shannon diversity on the other hand includes the detection probability and Simpson diversity goes even further and incorporates the squared detection probability. Therefore, species richness is best suited to address the diversity of rare species, Shannon diversity is best suited to address the diversity of frequent species and Simpson diversity is best suited to address the diversity of highly frequent species. In Chao et al. (2014, 2020) and Hill (1973) a more in-depth explanation is provided.

The calculation of the three chosen diversity measures followed the description by Colwell et al. (2012), Chao et al. (2020), and Nopp-Mayr et al. (2023) (for details and explanations of the procedure see also Online Resource, ESM 1, “Calculation methods”). To determine whether significant differences between exclosure and control plots existed 84% confidence intervals were calculated (Payton et al. 2003, Colwell et al. 2012). If the confidence intervals did not overlap a significant difference between the two groups was assumed. To define the number of bootstrap replications needed for the diversity analyses different amounts of replications were tested. At 1000 replications the calculated estimates stabilized, and this number of replications was henceforth used.

Additionally, we ran a Wilcoxon signed-rank test to compare the height of the tallest individual per tree species and plot type (open access vs. exclosure). To calculate the diversity measures the R packages “iNext” (Hsieh et al. 2016) and “iNext.4step” (Chao et al. 2020) were used. Graphs were created with the R package “ggplot2” (Wilkinson 2011).

## Results

### Tree species diversity

During the starting survey (year 0) a total of 18 different tree species were recorded on the 73 plot pairs that were monitored over 30 years (see Table 2). The survey in year 30 yielded 14 different tree species on the control plots and 13 on the exclosure plots. The minimum number of trees species detected on a single plot was 1 regardless of the treatment and the time of recording. The maximum number of species detected on a single plot however was different between years and treatment. On control plots, a maximum of 12 tree species in year 0 and 8 species in year 30 were detected (Table 2). In comparison, a maximum of 11 and 9 tree species was recorded on exclosure plots in year 0 and year 30, respectively. A list containing all detected species is available in the Online Resource (Table ESM 1).

**Table 2:** Total number of tree species detected on the 73 control and exclosure plots in the starting (year 0) and last survey (year 30). *n* = total number of species found on the 73 plots, min = minimum number of species detected on a single plot, max = maximum number of species detected on a single plot, median = median number of species detected on a single plot

	Control plots				Exclosure plots			
year	min	max	median	*n*	min	max	median	*n*
0	1	12	3	18	1	11	4	18
30	1	8	4	14	1	9	4	13

**Fig. 3:**
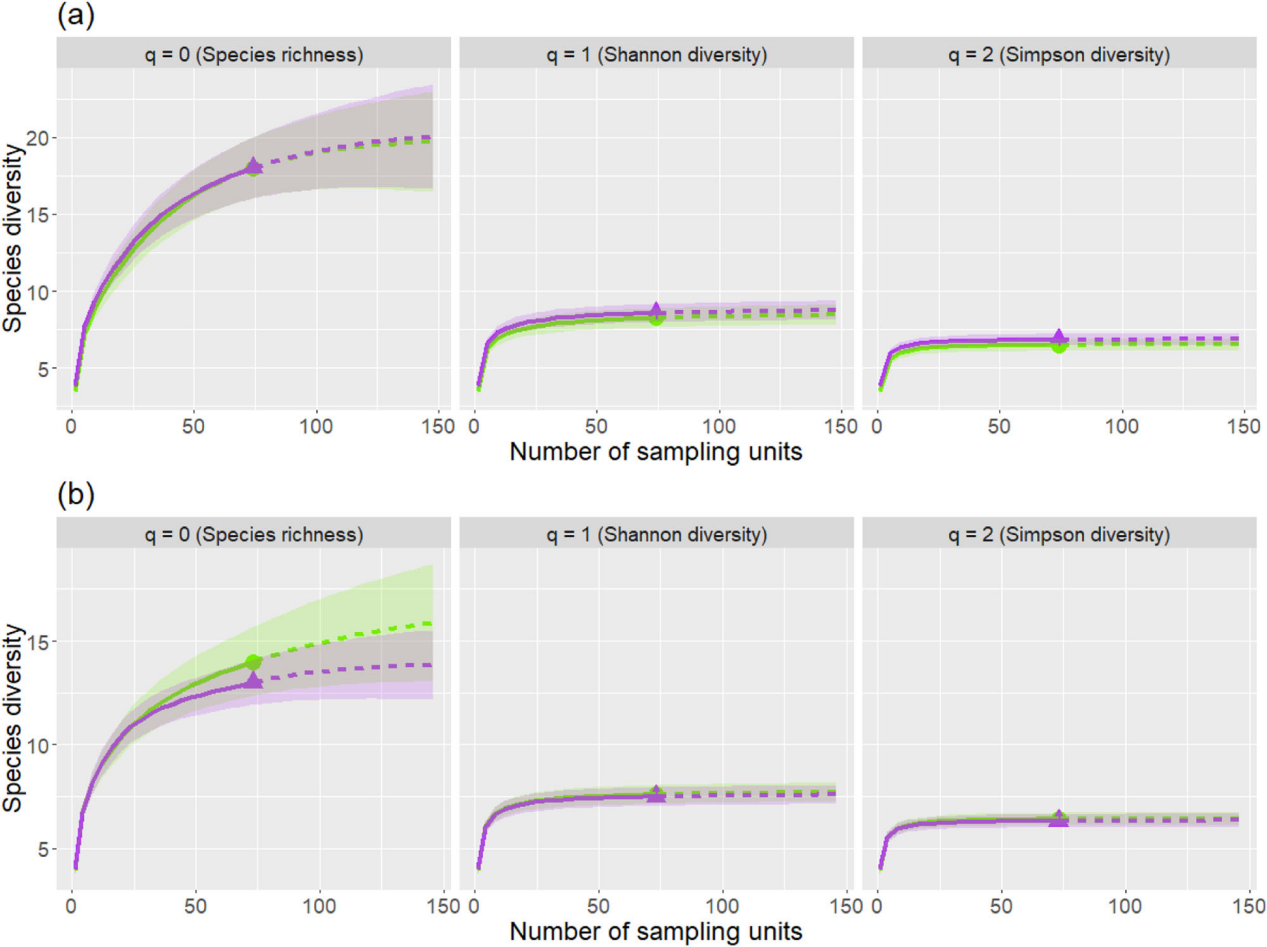
Empirical diversity profiles of sample size-based rarefaction (solid lines) and extrapolation (dashed lines) curves for **tree species diversity** in **a** the starting year ( = year 0) and **b** at year 30. Curves for control plots are depicted in green with points, whereas curves for fenced plots are depicted in purple with triangles. The 84% confidence intervals were calculated through bootstrapping based on 1000 replications. Please note the differently scaled *y*-axes for panel **a**, **b**

Sample size-based rarefaction and extrapolation sampling curves for Shannon diversity (*q* = 1) and Simpson diversity (*q* = 2) levelled off and approached an asymptote (see Fig. 3) for both control and exclosure plots in years 0 and 30. To infer “true” species richness (*q* = 0) non-asymptotic point diversity estimates had to be calculated at a standardized coverage value of C_max_ = 99.7% (see Online Resource, Table ESM 2).

For both control and fenced plots, the starting survey (year 0) yielded higher values for all three diversity measures than the last survey (year 30). Regardless of year, the 84% confidence intervals overlapped for all the diversity measures indicating no significant differences in tree species diversity between control plots and fenced plots.

### Height class diversity of trees

**Fig. 4:**
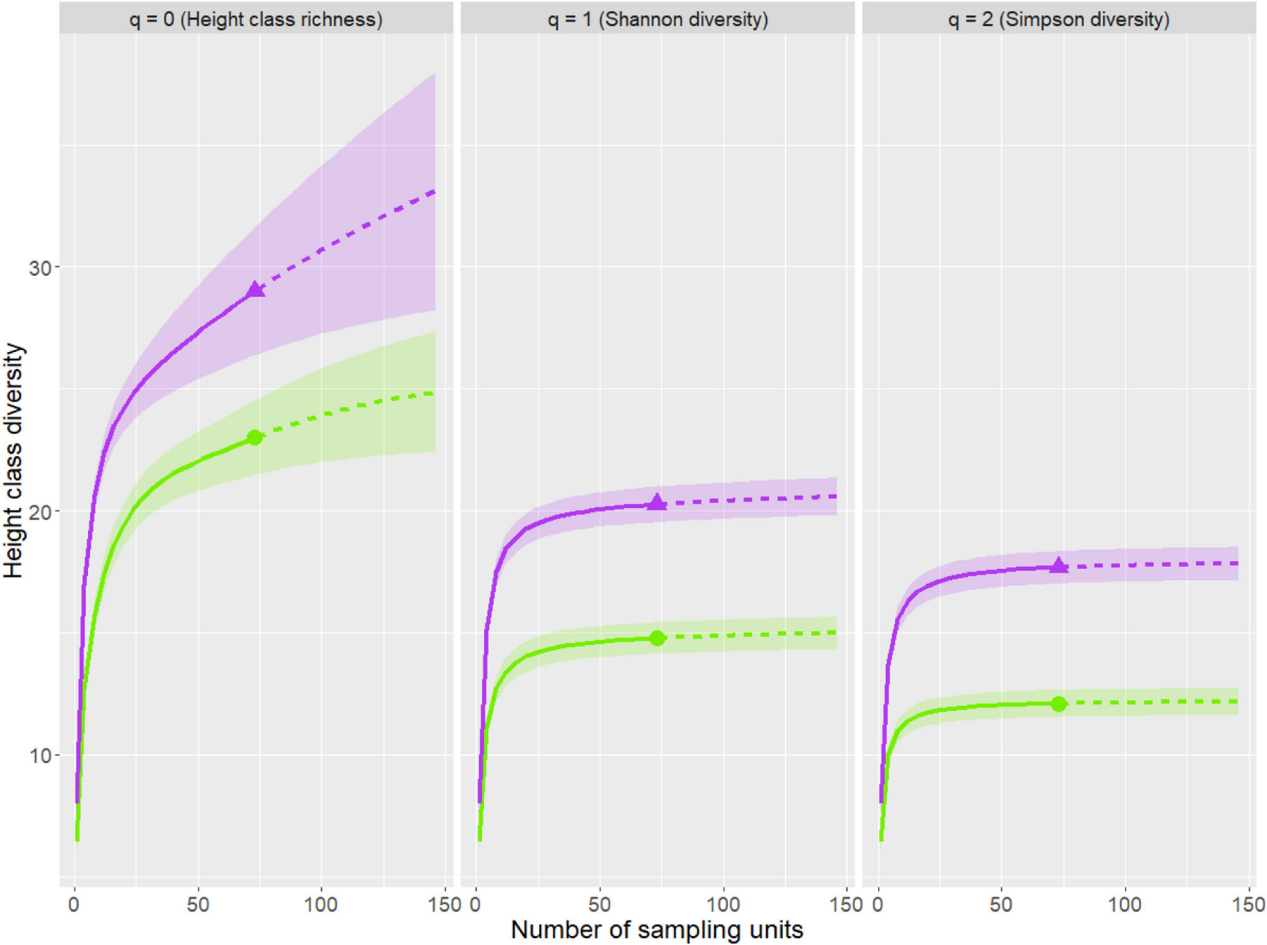
Empirical diversity profiles of sample size-based rarefaction (solid lines) and extrapolation (dashed lines) curves for **height class diversity** of the **tallest individuals** (n_max_ = 6) per tree species 30 years after establishment. Curves for control plots are depicted in green with points, whereas curves for fenced plots are depicted in purple with triangles. The 84% confidence intervals were calculated through bootstrapping based on 1000 replications

On the 73 plot pairs that were surveyed for 30 years, a total of 29 different height classes were recorded, of which 6 were only encountered on exclosure plots. A minimum of 2 and a maximum of 15 height classes were recorded on control plots, whereas a minimum of 3 and a maximum of 16 height classes were recorded on exclosure plots. Control plots yielded a median of 6.5 height classes per plot and exclosure plots a median of 8. Sample size-based rarefaction and extrapolation sampling curves for Shannon diversity (*q* = 1) and Simpson diversity (*q* = 2) approached an asymptote (see Fig. 4) for both control and exclosure plots. Contrary sample size was not large enough to infer “true” height class richness (*q* = 0) and the curves only represented a lower bound. To interpret height class richness non-asymptotic point diversity estimates at a standardized coverage value of C_max_ = 99.4% were calculated (see Online Resource, Table ESM 3).

Both Shannon diversity (*q* = 1) and Simpson diversity (*q* = 2) differed significantly between control and exclosure plots as indicated by non-overlapping confidence intervals. Frequent height classes were represented by a Shannon diversity of 20.57 in exclosure plots and 14.81 in control plots. The differences in Simpson diversity (17.83 in exclosure plots versus 12.10 in control plots) illustrated the impact of ungulate herbivory on highly frequent height classes. No significant differences in rare height classes between control and exclosure plots could be discovered as the confidence intervals of the height class richness overlapped.

### Height class distribution

**Fig. 5:**
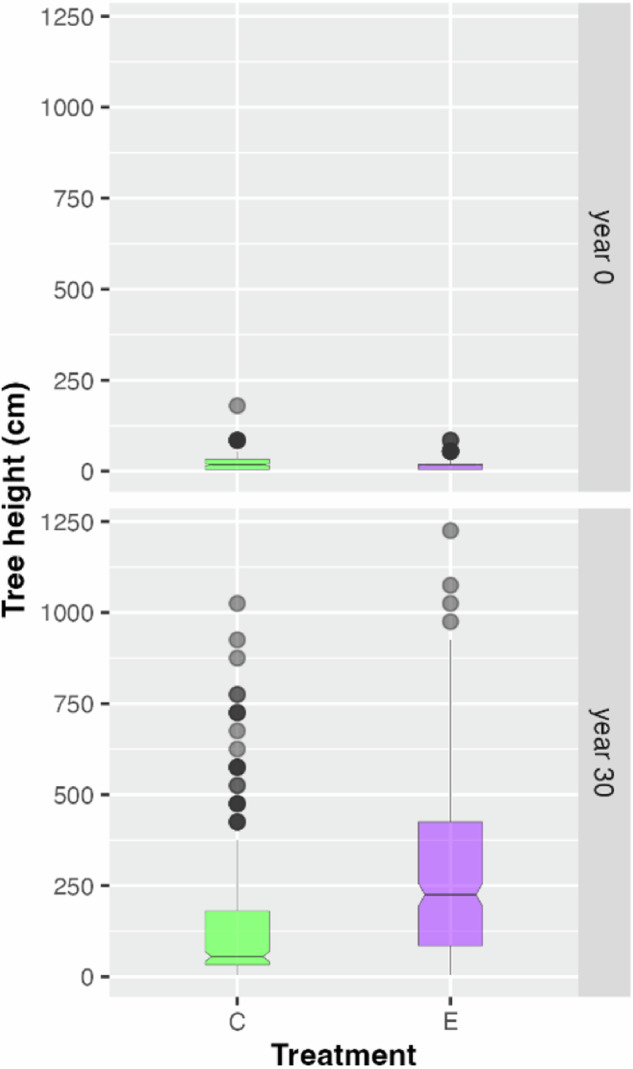
Boxplots of tree heights (cm) of the tallest individual per tree species recorded on control plots (“C”, green) and exclosure plots (“E”, purple) in the year of establishment (year 0, upper panel) and in year 30 (lower panel) after establishment and pooled over all tree species

**Fig. 6:**
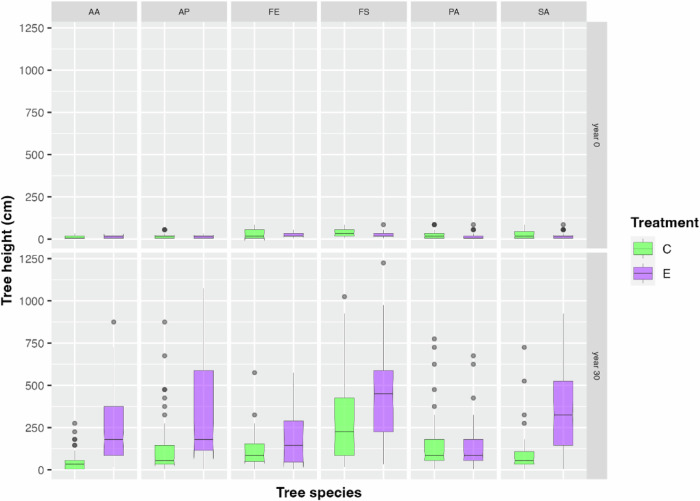
Boxplots of tree heights (cm) of the tallest individual per tree species recorded on control plots (“C”, green) and exclosure plots (“E”, purple) in the year of establishment (year 0, upper panel) and in year 30 (lower panel) after establishment. The most frequent species are depicted. Species abbreviations (the number of plots, where the species occurred, is given for year 0 and year 30 and for the control and exclosure plots C/E, respectively): AA *Abies* *alba* (35/36 and 45/48), AP *Acer pseudoplatanus* (35/36 and 45/48), FE *Fraxinus excelsior* (13/17 and 16/10), FS *Fagus sylvatica* (30/33 and 44/40), PA *Picea abies* (68/91 and 64/53), SA *Sorbus aucuparia* (31/39 and 42/53)

As differences in height class diversity do not inform about actual height growth differences between control and exclosure plots, we additionally contrasted the heights of the tallest individual per tree species and plot type (open access vs. exclosure) (Fig. 5). We used the mid-point height per height class interval as height representative for each tree (e.g., 85 cm for the 71–100 cm height class). A two-samples Wilcoxon test yielded significant height differences between control and exclosure plots in year 30 (*p* < 0.001) when pooling all tree species, reflecting generally suppressed height growth on the control plots. This delay can be observed in many frequently occurring, but not all tree species (Fig. 6), with Norway spruce being a clear exception (for a figure depicting all recorded tree species see Online Resource, Figure ESM 1).

### Shrub species diversity

We recorded 19 different shrub species on 59 out of 73 surveyed plot pairs in the course of the starting survey (year 0) (see Online Resource, Table ESM 4). On some plot pairs, no shrubs were detected.

In year 30, a total of 17 different shrub species were recorded. During the starting survey between 1 and 6 different shrub species were detected on control plots and between 1 and 5 on exclosure plots with the main shrub species being *Vaccinium myrtillus, Rubus idaeus, R. fruticosus* and *Lonicera sp*.. Control and exclosure plots both had a median of 2 shrub species. After 30 years, between 1 and 4 shrub species were detected on control plots and between 1 and 5 on exclosure plots. The median for control plots changed to 1.5 shrub species for control plots and stayed at 2 shrub species for exclosure plots.

**Fig. 7:**
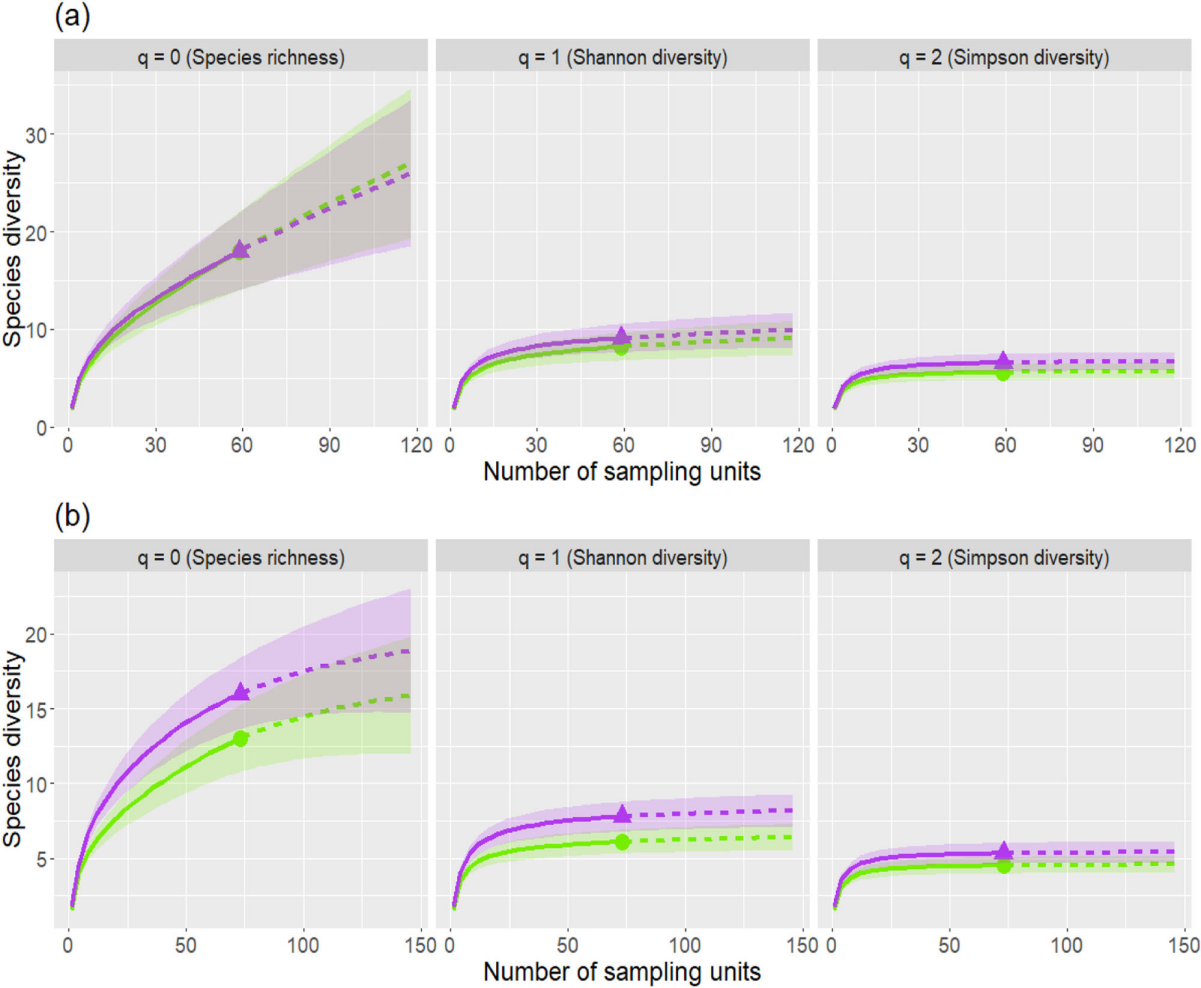
Empirical diversity profiles of sample size-based rarefaction (solid lines) and extrapolation (dashed lines) curves for **shrub species diversity** in **a** the starting year ( = year 0) and **b** at year 30. Curves for control plots are depicted in green with points, whereas curves for fenced plots are depicted in purple with triangles. **a** represents year 0, and **b** represents year 30. The 84% confidence intervals were calculated through bootstrapping based on 1000 replications. Please note the differently scaled y-axes for panel **a**, **b**

Sample size-based rarefaction and extrapolation sampling curves of Simpson diversity (*q* = 2) levelled off and approached an asymptote (see Fig. 7) for both control and exclosure plots in years 0 and 30. Shannon diversity (*q* = 1) only levelled off for the 30-year data thus highly frequent species with this method and the curves could be interpreted without additional measures. To infer “true” species richness (*q* = 1) and Shannon diversity (only for year 0) non-asymptotic point diversity estimates had to be calculated at a standardized coverage value of C_max_ = 92.1% (see Online Resource, Table ESM 5).

No significant differences in shrub species diversity were detected between control and exclosure plots in year 0 and year 30 as the confidence intervals overlapped for all diversity measures.

### Temporal pattern of the proportion of plots where terminal shoots outgrow the ungulate browsing height

#### Control plots vs. exclosures

**Fig. 8:**
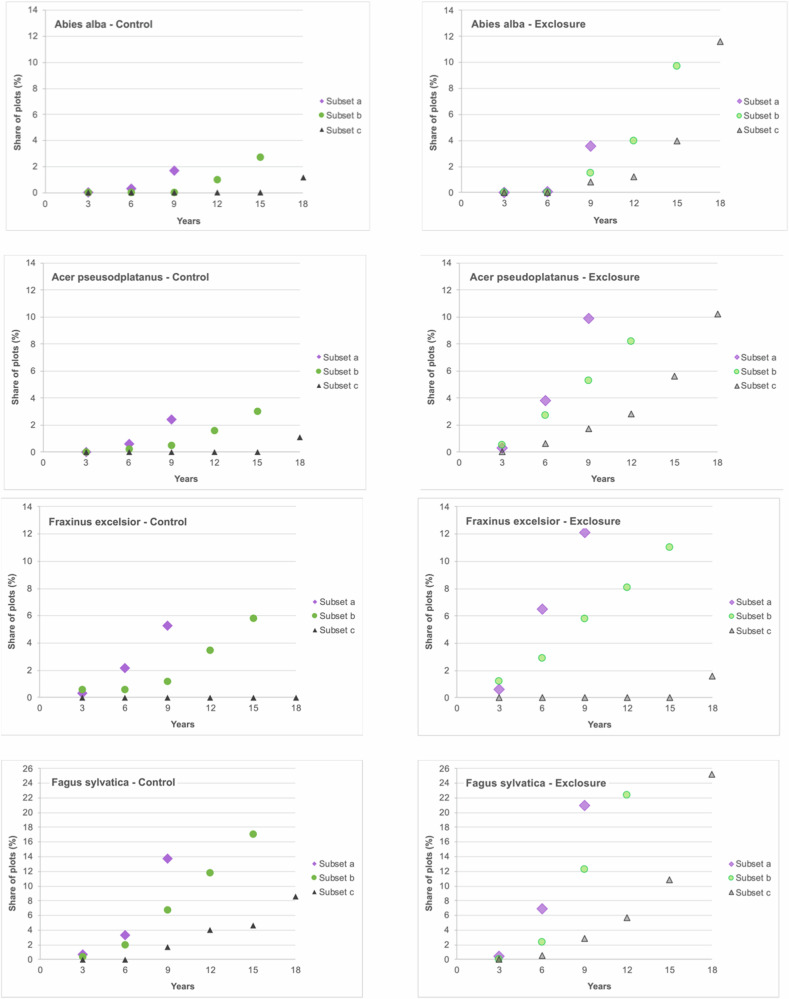

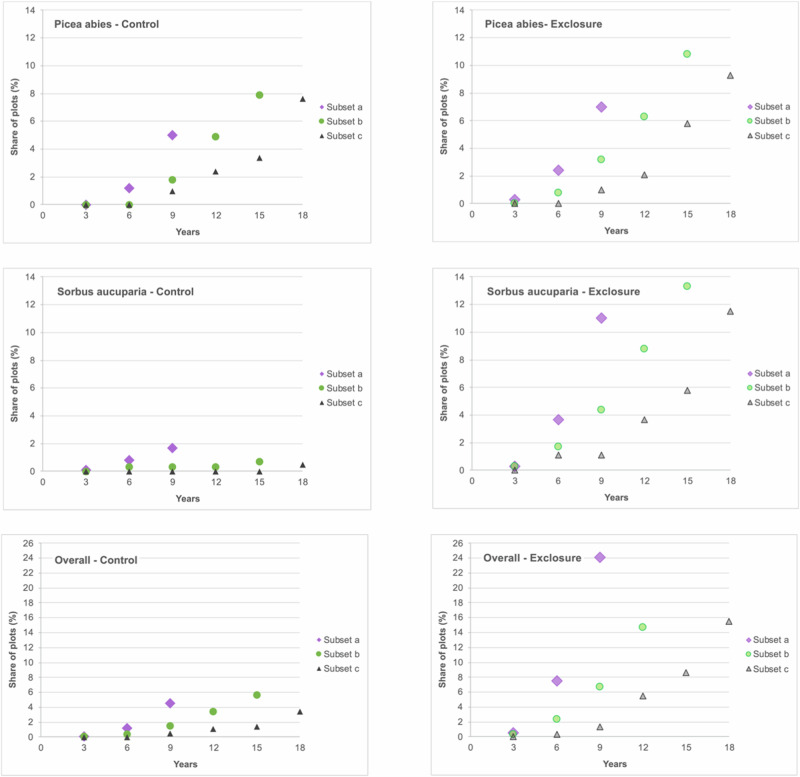
Share of plots, where a tree species reached a height of 160 cm in percent of the total number of plots, where the species occurred. Percentages are depicted for the control plots and the exclosures separately, for six observation periods (years) and for the three different subsamples, each including the plots depending on the minimum time span they were investigated (i.e., subset a: 9 years; subset b: 15 years; subset c: 24 years). The line *overall* represents the share of plots, where trees reached a height of 160 cm regardless of the tree species. Please note the different y-axis of the single graphs

To highlight potential differences between control and exclosure plots in the time the trees needed to outgrow ungulate browsing height, we used three subsamples of plot pairs that were surveyed in 3-year intervals for 9, 15 and 24 years. We defined height class 8 ( >160 cm) as related threshold, where trees´ terminal shoots are usually too high above ground to be accessed by ungulates under average conditions (i.e., outside periods with high and walkable snow coverage). In many cases, open access plots showed lower proportions and time delays until trees reached the 160 cm height threshold (Fig. 8). The delay was especially apparent in wild cherry (*Prunus avium*) and wych elm (*Ulmus glabra*) (see Online Resource, Table ESM 6). European beech (*Fagus sylvatica*), silver birch (*Betula pendula*), mountain ash (*Sorbus aucuparia*), and others showed distinctly lower percentages of plots where trees reached 160 cm tree heights. Within the frequently occurring tree species, the smallest differences between control and exclosure plots were observed for Norway spruce (*Picea abies*). It has to be mentioned that most tree species never reached height class 8 on most sites. This is also the reason why the percentages do not add up to 100.

#### Short-term vs. long-term survey duration

To address the question, whether long-term surveys might deviate from short-term records in terms of growth performance, we calculated the share of plots, where the occurring tree species reached a height of 160 cm in percent of the total number of surveyed plots, respectively. These percentages were depicted for three different subsets representing different investigation periods (subset a: 9 years; subset b: 15 years; subset c: 24 years). It became evident, that the portion of plots, where trees reached the height threshold, showed an earlier peak within the collective of plot pairs being surveyed for shorter periods of time (i.e., subset a vs. subset b vs. subset c; Fig. 8). This was the case both for open access and exclosure plots and also, when not particularly addressing a specific tree species, but any occurring tree species (see Fig. 8, panel “Overall”). Overall, on the 1722 plot pairs observed for at least 9 years (subset a) 7,5% of the exclosure plots and 1,2% of the control plots had at least one tree grown into the height class of 160 cm after 6 years of observation. Of the 332 plot pairs observed for at least 24 years (subset c) trees reached the height of 160 cm only on 0,5% of the exclosure plots and on none of the control plots after 6 years. The same pattern applies for single tree species. For example, after six years *Fagus sylvatica* on exclosure plots reached the benchmark of 160 cm ten times more often in subset a (6,9%) than in subset c (0,6%), indicating that subset c is driven by more slow-growing sites. The 50,9% of exclosure plots with outgrown beech trees after 24 years in subset c (Online Resource, Table ESM 6) do not reflect the true situation of growth rate in the whole area investigated, since the majority of plot pairs on faster-growing sites were already discarded earlier. These observations suggest that long-term survey data indeed differ in terms of height growth pattern. This means that lasting effects of ungulate impacts are reflected for specific situations, where long-term monitoring could be maintained, but not but not necessarily for the entire area of interest where monitoring takes place.

## Discussion

### Tree species diversity

Ungulates might impact the occurrence and abundance of plant species by feeding on available and preferred plants, thereby shaping plant communities and biodiversity at several scales. Vice versa, plant species exert specific strategies in responses to ungulate herbivory by adapting resistance and tolerance (Rhodes et al. 2017). Studies on effects of ungulate herbivory on tree species diversity provide largely divergent results: In some cases, ungulate herbivory resulted in higher tree species diversity (Nishizawa et al. 2016) whereas in other cases it led to a reduction (Bernes et al. 2018; Reimoser et al. 2022) or had no significant impact (Nopp-Mayr et al. 2023; Royo and Carson 2022). The latter case also applied to our 30-year data set, where no significant difference in diversity measures between open access plots and exclosures could be seen. There are many reasons for this huge bandwidth of effects. For example, different ungulate species show different feeding preferences (Ingram et al. 2013), which can tip the scales in interspecies competition between tree species (McGarvey et al. 2013; Weisberg and Bugmann 2003). This is particularly crucial for tree species which are very vulnerable to browsing and do not survive intense herbivory (Nishizawa et al. 2016). Highly preferred plant species might detract herbivores from other plant species, which in turn escape feeding pressure. Furthermore, rare plant species can be highly attractive for ungulates (Bödeker et al. 2021). If, however, a plethora of alternative feeding sources are available, vulnerable tree species might not be affected by herbivory (Kupferschmid et al. 2020). Different capabilities of plant species to produce toxins, thorns, and other herbivory deterrents plays a major role in the feeding decisions of ungulates. These capabilities, which sometimes are only produced if subjected to browsing by certain herbivores, are also influenced by environmental factors (Seager et al. 2013) or a plant´s life stage (Selaković et al. 2017). Furthermore, factors like the spatial and temporal distribution of ungulates, the applied silvicultural system, stem density of young trees and more also affect herbivore impacts on woody plants (Reimoser 2003). For example, Reimoser et al. (2022) found higher overall biodiversity in a large area in the case of an uneven spatial distribution of ungulates compared to evenly distributed or absent ungulates as high spatial variability in ungulate presence also leads to variability of related browsing exposure of plants.

The complexity of these driving factors including ungulate species composition or abundance, differences in environmental conditions (e.g., soil, aspect, or altitude), differences in plant communities together with differing time periods covered by surveys explains the variety of reported impacts of ungulate herbivory on tree species diversity. However, in contrast to the lack of effects within the pooled data set in our study (including all open access and exclosure plots at larger spatial scales), there might be distinct differences in tree species diversity between plot pairs on a local scale. Therefore, additional assessments for smaller areas with similar ecological conditions are recommended if management consequences are to be drawn from the effects of ungulates on tree species diversity.

Ungulates can impact forest vegetation by trampling (including pawing and scraping), fraying, bark-peeling and, perhaps most importantly, browsing (both visible browsing and unseen browsing, such as feeding on seeds and seedlings). Intense selective browsing of certain tree species often results in reduced regeneration of these species, thereby changing the overall tree species composition. However, such impacts do not automatically mean damage to the forest. In forestry, the occurrence of damage or benefit depends on the respective objective for the forest. Damage in forestry terms may arise if the ungulate impact leads to a reduction in forest growth, economic value, ecological stability, species diversity, or in a forest’s protective function (e.g. against avalanches, land-slides, erosion, floods) (Reimoser et al. 2023). Beneficial effects can result from increasing diversity and improved economic value by ungulates selectively browsing non-target plant species and by improving germination conditions (seed dispersal, impact of hooves, and droppings; cf. Gill and Beardall 2001; Howe and Westley 1988; Putman 1986, 1996; Reimoser 2003).

### Height class diversity of trees

The analysis of height class diversity as a proxy for vertical structural diversity of trees on the survey plots resulted in lower Shannon and Simpson diversity on control plots than on exclosure plots, indicating that ungulate herbivory reduced the vertical structural diversity. This result was in line with an earlier study from another Austrian alpine region (Nopp-Mayr et al. 2023) and might be partially due to the fact, that continuous browsing of the terminal shoot over long periods of time hampers height growth (Beguin et al. 2016). Therefore, most trees on control plots were attributed to a small number of different height classes in the given study. On exclosure plots, other factors than herbivory, including inter- and intraspecies competition combined with environmental factors might determine height growth patterns, which leads to a differentiation in vertical structure (Reimoser et al. 2023). Again, the multitude of co-occurring processes that shape height structures of plant communities including preferences or avoidance of specific plant species by ungulates (Burney and Jacobs 2011; Provenza et al. 1992, 2003), the duration and intensity of browsing, the seasonally changing food availability, or ungulates’ preferences for certain plant heights (Hansen and Clark 1977; Nelson and Leege 1982) might induce different modifications of structural features of plant communities by ungulate feeding. Additionally, plants try to vertically escape ungulate herbivory by height increment, by changing leaf traits (photosynthesis, morphology, and chemistry) at specific height strata (Rhodes et al. 2017; Sabo 2019) or plant architecture (Johnston et al. 2007), yielding non-uniform structural responses of plant communities to ungulate’s feeding. It should be mentioned that height classes in our study did not cover constant ranges, as smaller ranges were used for the first life stages of trees. This means that, while it is possible to draw conclusions on vertical structural diversity with height class diversity, it is not possible to infer on absolute height differences. With regard to achieving forestry goals in forest management, the differences observed in the diversity of vertical structure are unlikely to play a major role.

### Height class distribution of trees

As differences in absolute height growth, caused by ungulate herbivory, are potentially important for forest regeneration and later developmental phases, the heights of the six highest tree individuals per tree species and per plot were analyzed. As was to be expected, trees on exclosure plots were significantly higher compared to open control plots after a survey time of 30 years. The reason for this development over the 30 years is the repeated herbivory of ungulates, which reduces height growth, especially if terminal shoots are repeatedly browsed (Hood and Bayley 2009; Kupferschmid et al. 2019). This difference in height growth could have a particularly negative impact in our study area, as almost half of the state’s forests are protective forests against avalanches and rockfalls, where fast forest regeneration is of key importance. In such steep protective forests above settlements and roads, ungulates are hunted very intensively, if necessary, all year round.

While browsing of terminal shoots in a single year does not necessarily induce height growth suppression in individual trees, but might even promote height growth, longer lasting herbivory frequently results in a height growth delay of browsed trees (e.g., Kupferschmid et al. 2022; Siipilehto and Heikkilä 2005), lowering their competitive power (Kupferschmid et al. 2014). In our study, browsing of ungulates did not prevent forest trees from growing with time, but we observed a significantly differing height growth performance at year 30 of the trial.

### Shrub species diversity

In our study, overall shrub species diversity did not differ significantly between control and exclosure plots after a survey span of 30 years, which is in line with results from another long-term survey in the Austrian alps (Nopp-Mayr et al. 2023). However, earlier studies on the effects of ungulate herbivory on shrub species diversity yielded very different outcomes (see Bernes et al. 2018). Compared to other plant strata and taxa, deciduous woody plants show distinct seasonal changes in the amount of available biomass for large herbivore feeding, simultaneously being a crucial food resource during periods with snow cover (Cook 2002; Jenkins and Wright 1987). However, co-occurring woody plant species potentially compete for resources and intensive herbivore feeding on one species or specific individuals might shape inter- and intraspecific competition for the others (Krueger et al. 2009). Although herbivore´s preferences depend on the specific ecosystem context and the foraging types of ungulate species (see Shipley et al. 2009), they can be expected to have different effects of herbivory on the diversity of shrub species compared to tree species, but this was not the case in our study.

### Reaching the critical tree height - open access vs. exclosures and short-term vs. long-term surveys

Species-specific and context-dependent growth patterns of tree species (Rhodes et al. 2017) determine the time span, where terminal shoots of plants are accessible for ungulate herbivores as well as the chance for outgrow. The time it takes for trees to outgrow ungulate herbivory ties back into the afore-discussed height growth differences. Due to the slower growth on control plots, it takes trees longer to reach the 160 cm threshold with their terminal shoots than on exclosure plots. This is clearly expressed by a lower percentage of control plots where trees have grown out of most vulnerable height classes at a given time in the vast majority of cases (cf. Fig 8).

The reduction in height growth caused by ungulates was further cemented when comparing data sets with different survey duration. The longer our survey was running, the higher was the proportion of sites with lower height growth performance although the survey concept included trees being higher than the usual feeding height of wild ungulates. In other words, when comparing the share of plots where tree species reached the height threshold for a specific point of time (e.g., year 9 of survey), the short-term surveys yielded higher values compared to the data sets that covered long-term periods. This was the case both for control and exclosure plots. This indicates that height growth on long-standing plot pairs was slower than on plot pairs that did not stand for such long periods. Thus, long-term control-exclosure data sets indeed inherently carry a potential shift towards specific site conditions. This includes several factors, that drive the feasibility of long-term surveys and the number of plot pairs persisting over a time period of 30 years: (1) In mountainous terrain, natural disturbances like wind throws, avalanches and rockfall as well as material aging have a strong influence on the functional life of exclosures. This might strongly correlate with slope and other terrain features. Once the effectiveness of exclosures is impaired and ungulates gain access, the related plot pair must be abandoned. (2) Long-term comparisons between exclosures and open access plots require no or at least equal forest management in the immediate surroundings of individual plot pairs, which might not easily be ensured. In case that forest operations in the adjacent mature stand impact on of the two related plots in a different way, the plot pair has to be omitted. This also applies to natural abiotic disturbances. (3) Long-term surveys often cannot be carried out in a scientific or university context alone, but require the involvement of local stakeholders. This entails the risk that surveys are terminated once regeneration reaches a certain tree height. The current pressure of ungulates on forest regeneration is a common calculation basis for annual game harvest plans, which was also the case in our study. Therefore, plots that had outgrown 160 cm were of less interest for some practitioners within an ungulate impact monitoring scheme and some plot pairs had thus no longer been recorded or abolished. It must be said, however, that old slow-growing sites also show a skewed current impact of ungulates as they have been impacted by ungulate herbivory for many years prior and therefore are also a less reliable calculation basis for the new annual game harvest plans. It can be assumed, that all these factors have an influence on our data set although the magnitude of their impact cannot be easily delineated.

## Conclusion

The effects of ungulates on biodiversity of forest vegetation vary depending on the diversity indicators used. Basically, forest structure and dynamics respond to the density of ungulates as well as many other influencing factors in the forest environment. In order to filter out the lasting effects of ungulates from the entire complex of interacting impacts, long-term studies with ungulate exclusions are urgently required. Although the exclosures do not represent a natural situation by completely preventing access for ungulates, they are methodologically indispensable.

However, long-term studies over several decades have the disadvantage that the ungulate situation at the beginning of the monitoring can be completely different from that at the end of the study period, depending on the management measures taken. This makes the interpretation of the results more difficult. Additionally, there is a risk that at the end of the study, not all pairs of plots comparable to the initial situation will be available. If the plot-pairs that have been lost over the decades are not randomly distributed, the final results for the entire area to be studied are not directly comparable, as the selective loss of certain sample areas can lead to a shift of the results.

If long-term surveys are not carried out by scientists but by stakeholders, it must be ensured that the surveys are continued even if the time periods relevant to some specific management issues have already been exceeded. This is a crucial and challenging point in a long-term survey setting.

Short-term studies on the influence of ungulates on forest development have the advantage that they always refer to current ungulate populations, making them easier to interpret, and an efficient adaptive management is possible. Studies with only short-term monitoring results are also easier to finance. However, the long-term overall impact of ungulates on forest development with the complex compensation and reinforcement effects over time, remain unknown. If comparing studies with different durations, one should be aware of these pros and cons.

## Supplementary information

Supplementary Information

## Data Availability

Data will be provided in a publicly accessible repository (figshare.com), once the manuscript is accepted.
