# Supplementary material for: Long-term surveys of ungulates’ effects on tree and shrub species in mountainous forests –outcomes and potential limits
Source: Environ Manage. 2024 Oct 24;74(6):1190–206. doi: 10.1007/s00267-024-02063-4 (PMC11549197; doi:10.1007/s00267-024-02063-4)
Supplement: Supplementary file 1 — Supplementary Information [file 267_2024_2063_MOESM1_ESM.pdf]

Electronic Supplementary Material

Long-term surveys of ungulates' effects on tree and shrub species in mountainous forests – outcomes and potential limits

U. Nopp-Mayr[1*], W. Lechner[1a], S. Reimoser[2], F. Reimoser[1,2]

Calculation methods

The calculation of the three chosen diversity measures followed the description by Colwell et al. (2012), Chao et al. (2020), and Nopp-Mayr et al. (2023): The first step was the calculation of sample size-based rarefaction and extrapolation curves. The important role sample size plays in the detection and occurrence of species were the reason sample size-based curves were chosen. The extrapolation was used to double the sample size of surveyed fenced and control plots (Chao et al. 2014; Nopp-Mayr et al. 2023). (Chao et al. (2020) state that "true" diversity is observed if the sample size-based rarefaction and extrapolation curve approaches an asymptote. If this is not the case a comparison of diversity between the exclosure and control plots is not possible with this analysis. After calculating the curves, we assured that the data is sufficient. This was done via a visual examination of sample completeness profiles (Chao et al. 2020). As described in Nopp-Mayr et al. (2023) sample size-based rarefaction and extrapolation curves for Hill numbers of order q = 1 (Shannon diversity) and q = 2 (Simpson diversity) usually level off. This indicates that an interpretation of frequent and highly common species as well as a comparison between the assemblages is possible. The opposite is the case for Species richness (q = 0), for which in most cases the sample size-based rarefaction and extrapolation curves do not approach an asymptote. This means that the sample size is insufficient to interpret the rare species in an assemblage and the asymptotic estimator can only be interpreted as a lower bound of the true species diversity. To still be able to interpret such cases, a non-asymptotic standardization approach was utilized (Chao et al. 2020). The calculation of diversity estimates based on standardized sample coverage (maximum coverage C_{max}) allowed for a comparison between assemblages, even when the sample size-based rarefaction and extrapolation curves did not approach an asymptote (Chao et al. 2020).

Tree species diversity

Table ESM 1 Tree species present on at least one survey plot indicated by an "x". Separate columns for control plots and exclosure plots and the first survey (year 0) and the last survey (year 30).

	**Control plots**		**Exclosure plots**	
**Tree species**	Year 0	Year 30	Year 0	Year 30
Abies alba	x	x	x	x
Acer platanoides	x	x	x	x
Acer pseudoplatanus	x	x	x	x
Betula pendula	x	x	x	x
Fraxinus excelsior	x	x	x	x
Fagus sylvatica	x	x	x	x
Juglans regia	x	x	x	---
Larix decidua	---	---	x	---
Picea abies	x	x	x	x
Pinus sylvestris	x	---	x	---
Prunus avium	x	x	x	x
Quercus sp.	x	x	x	---
Sorbus aria	x	x	x	x
Sorbus aucuparia	x	x	x	x
Salix caprea	x	x	x	x
Salix sp.	x	---	---	---
Taxus baccata	x	---	x	x

Tilia sp.	x	---	x	---
Ulmus glabra	x	x	x	x

Table ESM 2 Asymptotic and non-asymptotic **tree species diversity** estimates for **year 0** and **year 30**. Non-asymptotic rarefaction and extrapolation analyses are based on point diversities for a specified sample coverage of C_{max} = 99.7 %. The Hill numbers of order q stand for species richness (q = 0), Shannon diversity (q = 1), and Simpson diversity (q = 2)

	**Control Plots**			**Exclosure Plots**		
Year	***q* = 0**	***q* = 1**	***q* = 2**	***q* = 0**	***q* = 1**	***q* = 2**
**Asymptotic diversity estimate (mean ± SE)**						
**0**	19.97 ± 5.34	8.55 ± 0.48	6.56 ± 0.28	20.63 ± 6.14	8.84 ± 0.47	6.89 ± 0.27
**30**	16.95 ± 4.06	7.77 ± 0.36	6.45 ± 0.21	13.98 ± 1.59	7.62 ± 0.33	6.36 ± 0.25
**Non-asymptotic point diversity estimate [84% confidence interval: lower; upper]**						
**0**	19.57 [12.8; 26.2]	8.42 [7.7; 9.0]	6.52 [6.1; 6.9]	20.04 [12.8; 27.2]	8.74 [8.1; 9.3]	6.86 [6.4; 7.2]
**30**	15.87 [11.5; 20.1]	7.70 [7.2; 8.1]	6.42 [6.1; 6.7]	13.4 [11.6; 15.2]	7.52 [7.0; 7.9]	6.32 [6.0; 6.6]

Height class diversity of trees

Table ESM 3 Asymptotic and non-asymptotic **height class diversity** estimates of the **tallest individuals** (n_{max} = 6) per tree species **30 years** after establishment. Non-asymptotic rarefaction and extrapolation analyses are based on point diversities for a specified sample of C_{max} = 99.4 %. The Hill numbers of order q stand for height class richness (q = 0), Shannon diversity (q = 1), and Simpson diversity (q = 2)

**Control Plots**			**Exclosure Plots**		
q = 0	q = 1	q = 2	q = 0	q = 1	q = 2
**Asymptotic diversity estimate (mean ± SE)**					
25.95 ± 2.41	15.11 ± 0.46	12.24 ± 0.39	41.32 ± 10.96	20.83 ± 0.60	17.98 ± 0.42
**Non-asymptotic point diversity estimate [84% confidence interval: lower; upper]**					
23.29 [20.6; 25.9]	14.81 [14.1; 15.4]	12.10 [11.5; 12.6]	33.10 [23.8; 42.3]	20.57 [19.6; 21.4]	17.83 [17.1; 18.5]

Height class distribution

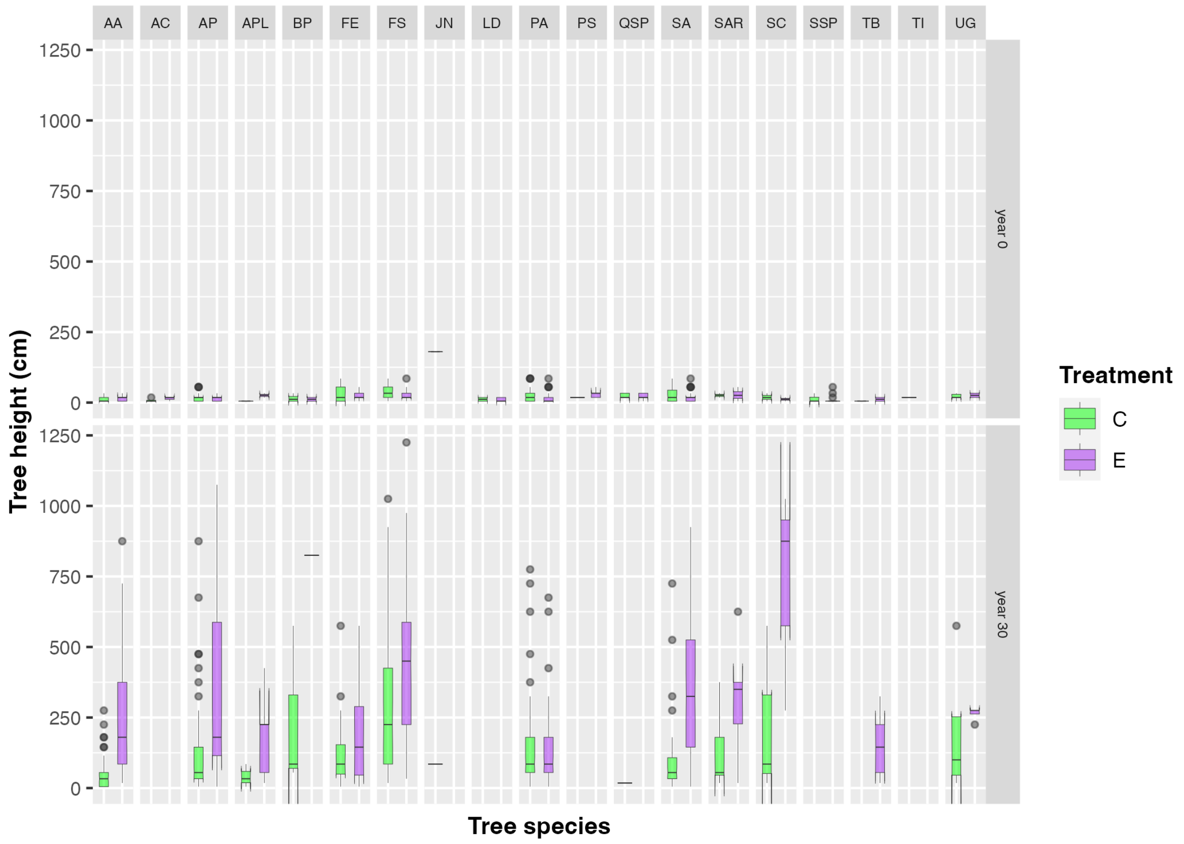

Fig. ESM 1 Boxplots of tree heights (cm) of the tallest individual per tree species recorded on control plots ("C", green) and exclosure plots ("E", purple) in the year of establishment (year 0, upper panel) and in year 30 (lower panel) after establishment. Species abbreviations (the number of plots, where the species occurred, is given for year 0 and year 30 and for the control and exclosure plots C/E, respectively): AA = *Abies* alba (35/36 and 45/48), AC = *Acer campestre* (4/3 and 0/0), AP = *Acer pseudoplatanus* (35/36 and 45/48), APL = *Acer platanoides* (2/2 and 3/5), BP = *Betula pendula* (4/4 and 3/1), FE = *Fraxinus excelsior* (13/17 and 16/10), FS = *Fagus sylvatica* (30/33 and 44/40), JN = *Juglans nigra* (1/0 and 1/0), LD = *Larix decidua* (8/9 and 0/0), PA = *Picea abies* (68/91 and 64/53), PS = *Pinus sylvestris* (2/5 and 0/0), QSP = *Quercus* sp. (5/9 and 1/0), SA = *Sorbus aucuparia* (31/39 and 42/53), SAR = *Sorbus aria* (2/4 and 8/8), SC = *Salix caprea* (3/2 and 3/3), SSP = *Salix* sp. (3/18 and 0/0), TB = *Taxus baccata* (1/4 and 0/5), TI = *Tilia* sp. (1/0 and 0/0), UG = *Ulmus glabra* (6/6 and 4/4)

Shrub species diversity

Table ESM 4 List of observed shrub species (in alphabetical order) on the control plots (C) and within the exclosures (E) in the year of establishment (year 0) and in year 30 after establishment

**Shrub species**	**Year 0**		**Year 30**	
	C	E	C	E
Rhamnus frangula	x	x	---	x
Berberis vulgaris	x	x	---	---
Vaccinium myrtillus	x	x	x	x
Sambucus nigra	x	x	x	---
Rubus fruticosus	x	x	x	x
Rhamnus sp.	---	x	---	---
Cornus sanguinea	x	x	---	x
Calluna vulgaris	---	---	---	x
Daphne sp.	x	x	x	x
Ilex aquifolium	x	---	x	x
Hedera helix	x	x	x	x
Corylus avellana	x	x	x	x
Lonicera sp.	x	x	x	x
Vaccinium vitis-idaea	---	---	---	x
Ligustrum vulgare	x	x	x	x
Rubus idaeus	x	x	x	x
Sambucus racemosa	x	x	x	x
Rosa sp.	x	x	x	x
Euonymus europaeus	x	x	---	---
Viburnum lantana	x	x	x	x

Table ESM 5 Asymptotic and non-asymptotic **shrub diversity** estimates for **year 0** and **year 30**. Non-asymptotic rarefaction and extrapolation analyses are based on point diversities for a specified sample coverage of C_{max} = 92.1%. The Hill numbers of order q stand for species richness (q = 0), Shannon diversity (q = 1), and Simpson diversity (q = 2)

	**Control Plots**			**Exclosure Plots**		
Year	q = 0	q = 1	q = 2	q = 0	q = 1	q = 2
**Asymptotic diversity estimate (mean ± SE)**						
0	67.15 ± 33.03	10.36 ± 1.91	5.80 ± 0.61	57.81 ± 25.26	11.01 ± 1.59	6.85 ± 0.66
30	17.10 ± 5.85	6.58 ± 0.62	4.63 ± 0.41	20.10 ± 8.60	8.42 ± 0.75	5.47 ± 0.52
**Non-asymptotic point diversity estimate [84% confidence interval: lower; upper]**						
0	27.03 [1.7; 52.2]	9.07 [6.3; 11.7]	5.69 [4.8; 6.5]	18.53 [2.0; 35.0]	9.14 [6.7; 11.5]	6.58 [5.5; 7.5]
30	15.87 [7.7; 24.0]	6.39 [5.4; 7.3]	4.57 [4.0; 5.1]	18.7 [10.2; 27.1]	8.15 [7.0; 9.2]	5.39 [4.6; 6.0]

Table ESM 6 Share of plots, where tree species reached a height of 160 cm in percent of the total number of plots (n). n represents all plots where the tree species occurred either on the open access control plot (Var C) or within the exclosure (Var E). Percentages are depicted for the eight observation periods (years) and for three different subsamples, each including the plots depending on the minimum time span they were investigated (i.e., subsample a: 9 years; subsample b: 15 years; subsample c: 24 years). The line *overall* represents the share of plots, where trees reached a height of 160 cm regardless of the tree species.

Tree species	Var	Observation period (years)																		
		3			6			9			12		15		18	21	24	*n*		
		a	b	c	a	b	c	a	b	c	b	c	b	c	c	c	c	*a*	*b*	*c*
Abies alba	C	0,0	0,0	0,0	0,3	0,0	0,0	1,7	0,0	0,0	1,0	0,0	2,7	0,0	1,2	2,8	3,6	*1130*	*621*	*241*
	E	0,0	0,0	0,0	0,1	0,0	0,0	3,6	1,5	0,8	4,0	1,2	9,7	4,0	11,6	17,9	25,1			
Acer platanoides	C	0,0	0,0	0,0	0,0	0,0	0,0	0,0	0,0	0,0	0,0	0,0	0,0	0,0	0,0	0,0	0,0	*64*	*36*	*15*
	E	0,0	0,0	0,0	0,0	0,0	0,0	3,1	0,0	0,0	0,0	0,0	2,8	0,0	0,0	6,7	13,3			
Acer pseudoplatanus	C	0,0	0,0	0,0	0,6	0,2	0,0	2,4	0,5	0,0	1,6	0,0	3,0	0,0	1,1	2,3	6,2	*790*	*439*	*177*
	E	0,3	0,5	0,0	3,8	2,7	0,6	9,9	5,3	1,7	8,2	2,8	14,6	5,6	10,2	16,9	26,0			
Betula pendula	C	0,0	0,0	0,0	1,8	0,0	0,0	7,0	0,0	0,0	1,8	0,0	1,8	0,0	0,0	9,5	9,5	*114*	*57*	*21*
	E	0,0	0,0	0,0	4,4	1,8	0,0	13,2	1,8	0,0	3,5	0,0	5,3	0,0	0,0	19,1	33,3			
Fraxinus excelsior	C	0,3	0,6	0,0	2,2	0,6	0,0	5,3	1,2	0,0	3,5	0,0	5,8	0,0	0,0	0,0	3,2	*323*	*173*	*63*
	E	0,6	1,2	0,0	6,5	2,9	0,0	12,1	5,8	0,0	8,1	0,0	11,0	0,0	1,6	3,2	11,1			
Fagus sylvatica	C	0,7	0,4	0,0	3,3	2,0	0,0	13,7	6,8	1,7	11,8	4,0	17,1	4,6	8,6	15,4	18,9	*872*	*456*	*175*
	E	0,5	0,0	0,0	6,9	2,4	0,6	21,0	12,3	2,9	22,4	5,7	34,2	10,9	25,2	40,6	50,9			
Picea abies	C	0,0	0,0	0,0	1,2	0,0	0,0	5,0	1,8	1,0	4,9	2,4	7,9	3,4	7,6	11,3	15,1	*1216*	*668*	*291*
	E	0,3	0,0	0,0	2,4	0,8	0,0	7,0	3,2	1,0	6,3	2,1	10,8	5,8	9,3	12,7	18,2			
Prunus avium	C	0,0	0,0	0,0	0,0	0,0	0,0	0,0	0,0	0,0	0,0	0,0	5,6	0,0	0,0	0,0	0,0	*43*	*18*	*5*
	E	0,0	0,0	0,0	4,7	0,0	0,0	9,3	0,0	0,0	0,0	0,0	5,6	0,0	0,0	0,0	20,0			
Quercus sp,	C	0,0	0,0	0,0	0,0	0,0	0,0	1,2	0,0	0,0	0,0	0,0	0,0	0,0	0,0	0,0	0,0	*163*	*81*	*31*
	E	0,0	0,0	0,0	0,0	0,0	0,0	4,9	0,0	0,0	0,0	0,0	2,5	0,0	0,0	0,0	3,2			
Sorbus aria	C	0,0	0,0	0,0	0,0	0,0	0,0	0,9	0,0	0,0	0,0	0,0	1,3	0,0	0,0	8,3	11,1	*115*	*76*	*36*
	E	0,0	0,0	0,0	0,0	0,0	0,0	1,7	0,0	0,0	1,3	0,0	4,0	0,0	0,0	5,6	16,7			

Continuation of Table ESM 6 Share of plots, where tree species reached a height of 160 cm in percent of the total number of plots (n). n represents all plots where the tree species occurred either on the open access control plot (Var C) or within the exclosure (Var E). Percentages are depicted for the eight observation periods (years) and for three different subsamples, each including the plots depending on the minimum time span they were investigated (i.e., subsample a: 9 years; subsample b: 15 years; subsample c: 24 years). The line *overall* represents the share of plots, where trees reached a height of 160 cm regardless of the tree species.

Sorbus aucuparia	C	0,1	0,0	0,0	0,8	0,3	0,0	1,7	0,3	0,0	0,3	0,0	0,7	0,0	0,5	0,5	2,1	*728*	*407*	*191*
	E	0,3	0,3	0,0	3,7	1,7	1,1	11,0	4,4	1,1	8,8	3,7	13,3	5,8	11,5	23,0	33,5			
Salix caprea	C	0,0	0,0	0,0	0,0	0,0	0,0	1,4	0,0	0,0	0,0	0,0	0,0	0,0	14,3	14,3	14,3	*70*	*29*	*7*
	E	1,4	0,0	0,0	1,4	0,0	0,0	7,1	0,0	0,0	10,3	14,3	20,7	14,3	14,3	14,3	14,3			
Salix sp,	C	0,0	0,0	0,0	2,7	0,0	0,0	2,7	0,0	0,0	0,0	0,0	0,0	0,0	0,0	0,0	12,5	*37*	*17*	*8*
	E	0,0	0,0	0,0	2,7	0,0	0,0	8,1	0,0	0,0	0,0	0,0	0,0	0,0	0,0	0,0	25,0			
Taxus baccata	C	0,0	0,0	0,0	0,0	0,0	0,0	0,0	0,0	0,0	0,0	0,0	0,0	0,0	0,0	0,0	0,0	*61*	*35*	*18*
	E	0,0	0,0	0,0	0,0	0,0	0,0	0,0	0,0	0,0	0,0	0,0	2,9	0,0	0,0	0,0	0,0			
Ulmus glabra	C	0,0	0,0	0,0	0,0	0,0	0,0	0,0	0,0	0,0	0,0	0,0	3,9	0,0	0,0	0,0	5,9	*100*	*52*	*17*
	E	0,0	0,0	0,0	2,0	0,0	0,0	12,0	0,0	0,0	1,9	0,0	5,8	0,0	0,0	11,8	17,6			
**Overall**	**C**	**0,1**	**0,1**	**0,0**	**1,2**	**0,4**	**0,0**	**4,5**	**1,5**	**0,5**	**3,4**	**1,1**	**5,7**	**1,4**	**3,4**	**6,0**	**8,6**	***1722***	***907***	***332***
	**E**	**0,6**	**0,4**	**0,0**	**7,5**	**2,4**	**0,4**	**24,1**	**6,7**	**1,4**	**14,7**	**5,5**	**28,0**	**8,6**	**15,5**	**31,9**	**57,3**			
